# Long-Term Influence of Platelet-Rich Plasma (PRP) on Dental Implants after Maxillary Augmentation: Retrospective Clinical and Radiological Outcomes of a Randomized Controlled Clinical Trial

**DOI:** 10.3390/jcm9020355

**Published:** 2020-01-28

**Authors:** Sameh Attia, Clara Narberhaus, Heidrun Schaaf, Philipp Streckbein, Jörn Pons-Kühnemann, Christian Schmitt, Friedrich Wilhelm Neukam, Hans-Peter Howaldt, Sebastian Böttger

**Affiliations:** 1 Department of Cranio Maxillofacial Surgery, Justus-Liebig University Giessen, Klinik Str. 33, 35392 Giessen, Germany; 2 Medical Statistics, Institute for Medical Informatics, Faculty of Medicine, Justus-Liebig University Giessen, Rudolf-Buchheim Str. 6, 35392 Giessen, Germany; 3 Department of Oral and Maxillofacial Surgery, University of Erlangen, Glückstr. 11, 91054 Erlangen Germany

**Keywords:** dental Implant, PRP, Long-term result, Sinus lift, clinical outcome, radiological outcome

## Abstract

The long-term clinical and radiological outcomes of dental implants inserted in augmented bone treated with platelet-rich plasma (PRP) has not been well addressed in the literature yet. This study is based on a collection of patients from a randomized controlled trial (RCT) that did not report any short-term positive effects of PRP on bone healing after sinus lift surgery using autologous iliac crest bone graft. This study aimed to evaluate the long-term impact of PRP regarding clinical and radiological outcomes on the inserted implants in the previous RCT. For this evaluation, we considered the following variables: plaque index, probing depth, bleeding index, mobility grade, Periotest^®^ values, and radiological bone loss. Out of 53 patients (*n* = 306 implants) included in the previous study we were able to reinvestigate 37 patients (*n* = 210 implants) in two centers (31 in Giessen, Germany and 6 in Erlangen, Germany). Clinical and radiographic parameters suggested overall healthy conditions of the peri-implant tissue. The PRP-group and the control group did not differ significantly in the majority of the parameters. The overall evaluation showed that result data of the PRP-group was inferior to the control group in 64 percent of the evaluated parameters. The present study cannot provide evidence of a positive effect of PRP on the long-term implant clinical and radiological outcomes. In fact, a tendency towards inferior long-term results in the PRP-group was detected without reaching a significant threshold. Further controlled trials need to be conducted to investigate this correlation.

## 1. Introduction

Dental implants have high survival probabilities with good clinical and radiological findings [1]. Long-term studies documented a survival rate of approximately 95% over a study period of at least ten years [2,3]. One of the important factors leading to late implant loss is peri-implantitis, which is a combination of mucositis and bone resorption around implants [4,5,6]. Pain, implant mobility, crestal bone loss over a half of implant length, and persistent exudate from the peri-implant tissue are the most common reasons for implant removal [7]. Studies have reported a correlation between autologous cancellous bone grafts and long-term bone resorption, which leads to peri-implantitis and consecutive implant loss [8,9].

Bone loss resulting from trauma, disease, aging, or congenital abnormalities, remains a global challenge for health professionals and patients. Maxillofacial or craniofacial bone loss has a high psychological impact on patients. Therefore, aesthetic reconstruction is as valuable as functional reconstruction in this region [10]. 

Tissue engineering is a feasible method which can be pursued to perform aesthetic reconstruction. This implies the enhancement of autologous bone grafts using PRP [11]. In recent years, a staggering development on the use of tissue engineering and biomaterials has shifted beliefs and expectations [12]. Although recent strategies, such as decellularized matrix, nanoparticles, stem-cell therapies, scaffolds, and the engineering of a whole tooth, have shown some success, older approaches such as platelet-rich plasma (PRP) and platelet-rich fibrin (PRF) still require validation or disproof of their clinical efficacy through long-term follow-ups [12,13,14].

Since the first introduction of the use of PRP in augmentation techniques [11], various studies have been conducted to evaluate its effectiveness [15,16,17,18,19,20]. Optimized bone healing with enhanced bone mineral density was the most tested hypothesis in these studies. Results were controversial, with positive effects [15,18] and no effects [17,21] of PRP. Following initial positive effects of PRP, Consolo et al. reported a decrease of the bone mineral density after six months [18]. In another study, the same period was needed to report the first improvement of bone density by using PRP [22]. The fact is, if the bone density improves after augmentation using a mainly cancellous bone graft, such from the iliac crest bone, the long-term bone resorption around dental implants will be reduced [23]. Although the literature has addressed augmentation with and without PRP, long-term follow up has been rarely discussed. Thor et al. compared implant survival, marginal bone level, and implant stability after one year of mastication function of inserted dental implants in the maxilla, following autogenous bone grafting with or without PRP in a split mouth design [24]. The implant survival and marginal bone level revealed no statistically significant differences. Implant stability was tested by using resonance frequency analysis (RFA). Only implants in the anterior maxilla proved to be more stable on PRP sites [24]. Recent studies focused on the effect of PRP have had some limitations, such as a lack of a control group or only mid-term follow-up time [25,26,27]. Clinical practitioners judge the validity and feasibility of a given procedure more through a randomized controlled trial with long-term results. To the best of our knowledge, long-term clinical and radiological implant assessments after using an autologous iliac crest bone graft and PRP have not been well addressed in the literature. To investigate the long-term influence of PRP, we used the same collection of patients from the prospective randomized clinical trial (RCT) by Schaaf et al. [19]. The RCT was conducted between 2001 and 2004 in two centers (Oral and Maxillofacial Departments at the Universities of Giessen and Erlangen, Germany). 53 Patients (34 split-mouth, 19 unilateral) with maxillary atrophy underwent sinus lift surgery and iliac crest augmentation with and without PRP. That study could not determine any significant differences between the test and control group regarding bone density [19]. In this study, we hypothesized that dental implants placed in PRP-augmented bones have better clinical and radiological outcomes.

## 2. Materials and Methods

### 2.1. Study Design and Patient Groups

The study has been designed as a randomized controlled single-blind retrospective study. 53 patients from the Schaaf et al. study were included in this retrospective clinical and radiological study. The majority of the patients were examined in the Department of Cranio-Maxillofacial surgery in the University Hospital of Giessen. In addition, some examinations were carried out at the University Hospital in Erlangen. Due to the long journey from their place of residence, some patients were examined at the offices of their family dentists. Nevertheless, all examinations were carried out by the same evaluator. Patients were divided into two groups: split-mouth and unilateral groups to make the results comparable. Pregnancy and bad general condition were the exclusion criteria. Examiner preparation was done before starting the follow-up investigation. The evaluator prepared by investigating 50 dental implants clinically and radiologically from randomly selected patients. None of these patients were part of the study. Independently, an experienced oral surgeon evaluated the same implants and patients. The results of both evaluators were then compared. Agreement between the evaluators was measured by Kappa and Bland-Altman analysis. For the plaque index, Kappa values were 0.940 (*p* ≤ 0.001), for probing depth 0.870 (*p* ≤ 0.001), for bleeding index 0.919 (*p* ≤ 0.001), and the Periotest^®^ was 1.000 (*p* ≤ 0.001). In the mobility test, no variation was detected, therefore no Kappa calculation was possible. Agreement of bone loss measurement was proven by Bland-Altman analysis. We detected a bias of 0.04 with limits of agreement from -0.6 to 0.52. This calibration method was aimed at improving the accuracy of the diagnostic tasks as previously reported and recommended [28]. Radiographs were evaluated independently in a random fashion by the evaluator and an experienced oral surgeon to avoid investigation bias. The current study re-examined patients who participated in a randomized controlled prospective study after a mean follow-up period of 13 years. In this follow-up study, the single evaluator of the clinical examination and both evaluators of the radiological examination were blinded to the treatment with PRP. A number of 23 patients were treated bilaterally in a split-mouth design and 14 patients were treated on one side only. The patients who were treated unilaterally were randomly assigned to the study or control group depending on the side treated. The bilaterally treated patients were classified to the following subgroups “right with PRP, left without PRP” or “right without PRP, left with PRP “. This was done with adaptive stratification using central telephone randomization for both treatment centers. This study follows the CONSORT 2010 checklist developed for reporting a randomized trial [29].

### 2.2. Measured Variables 

#### 2.2.1. Clinical Parameters:

Clinical parameters used to evaluate dental implants include the following.

##### Plaque Index

To document the plaque colonization on the implant, the Mombelli plaque index was used with these characteristics:

Grade 0: No plaque detectable
Grade 1: Plaque only detectable by probing
Grade 2: Visible plaque deposit
Grade 3: Massive plaque deposition [30].

##### Probing Depth

The probing depths around the implants were measured at four sites (mesial, vestibular, distal, and palatal) using a plastic Click Probe ™ (Kerr Dental, Bioggio, Switzerland).

##### Bleeding Index

The bleeding tendency of the gingiva during probing was measured parallel to the probing depths. Existing or missed bleeding was also documented at four sites per implant.

##### Mobility Grade

The degree of clinical loosening was determined for each implant. The different grades were categorized into the following grades:

Grade 0: No increased mobility
Grade 1: Perceptible and visible horizontal mobility ≤1 mm
Grade 2: Visible horizontal mobility >1 mm
Grade 3: Movement through tongue or lip pressure.

##### Periotest^®^ Values

The Periotest^®^ Classic device (Medizintechnik Gulden, Modautal, Germany) can be used to evaluate the osseointegration of implants [31]. The device taps the implant with a plunger and measures how much the plunger is dampened by the implant. The results are classified into the following values:

−8 to 0 Satisfactory osseointegration
+1 to +9 Clinical examination of the implant required
+10 to +50 Insufficient osseointegration.

#### 2.2.2. Radiological Parameters:

**Figure 1:**
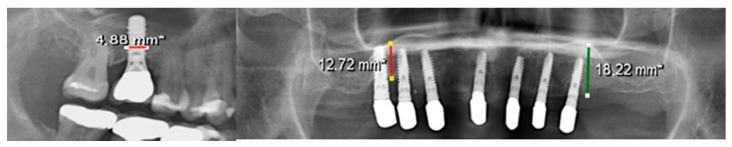
Calibration of the panoramic X-ray for accurate measurement of the bone resorption. *: calibrated according to scale bar.

Bone loss of the augmented bone region in the upper jaw was also determined. The alveolar crest height in the upper jaw was measure in a panoramic X-ray. The difference in bone resorption was determined using the post-augmentation alveolar crest height in the previous study. The Sidexis 4 Viewer^®^ program was used to measure the alveolar ridge height. The measurement was performed as carried out in the study by Schaaf et al., right and left upper second premolar and first molar. To eliminate the magnification factors in the panoramic X-ray, calibration using the implant diameter was used (Figure 1). Subsequently, the distance from the sinus floor to the alveolar crest was measured in second premolar and first molar regions and calculated with the previously calculated magnification factor. To calculate bone loss, the calculated alveolar crest height was subtracted from the value measured in the previous study prior to implant surgery. 

**Smoking**

Patients should provide information on their smoking behavior in the following increments:

- 1–5 cigarettes a day
- 5–15 cigarettes a day
- > than 16 cigarettes a day.

### 2.3. Statistical Analysis

Data recorded from the patients were divided to unilateral and bilateral groups of patients and were analyzed separately. The evaluation of the clinical and radiological parameters was performed mainly on the inserted implant in the two groups. A Fischer’s exact test or a chi-square test was used to analyze the qualitative variables. Quantitative variables were compared using the Mann-Whitney and Wilcoxon rank sum test. SPSS 25 was used for data analysis (IBM Corp. Released 2017. IBM SPSS Statistics for Windows, Version 25.0. Armonk, NY: IBM Corp.). Normality distribution of quantitative variables was verified by graphical methods (Histogramm, QQ-Plots).

### 2.4. Ethics Approval

This study was approved by the ethical committee of the medical faculity at Justus-Liebig University of Giessen (Approval number 129/15).

## 3. Results

**Figure 2:**
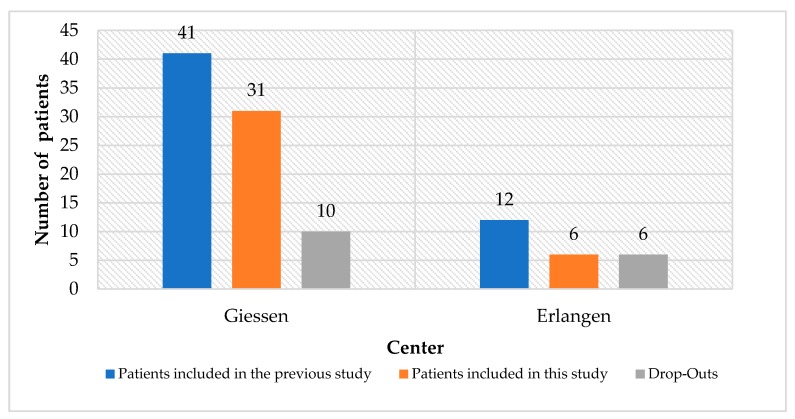
Number of patients included in this study versus number of drop-outs in the two clinics.

Out of 53 patients (*n* = 306 implants) included in the previous study by Schaaf et al., we were able to investigate 37 patients (*n* = 210 implants) in two centers (31 in Giessen, Germany and 6 in Erlangen, Germany). Various reasons lead to the drop-out of 16 patients such as: deceased or bad physical condition (*n* = 4), rejection of participation (*n* = 7), and patients not reachable (*n* = 5) (Figure 2).

25 female (67.6%) and 12 male (32.4%) patients with ages ranging between 30 and 90 years (median 65 years) and 210 implants were included. Out of 210 inserted dental implants, eight implants were lost and removed. Time between implant placement and follow-up examination ranged between 11.3 and 14.1 years (mean 13 years). The survival rate for all inserted implants was 96.2%. The statistical analysis was divided into two evaluation groups depending on the treatment design. Patients who were treated bilaterally (*n* = 23) were included in the split-mouth evaluation. The unilateral evaluation included the unilaterally treated patients (*n* = 14) and the 23 patients from the split-mouth evaluation, which were randomly included with one side of the body in the evaluation, so that the participants belonged to either the PRP or the control group. 

### 3.1. Split-Mouth Evaluation

**Figure 3:**
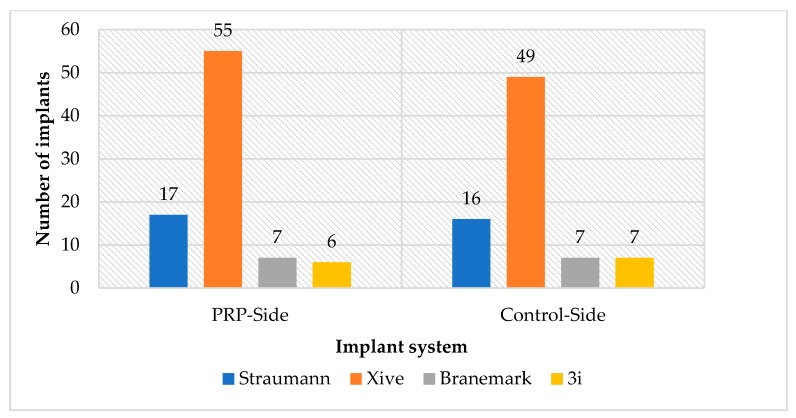
Number of implant in each implant system in both the platelet-rich plasma (PRP) and control sides.

All 23 patients who were treated bilaterally, one side with PRP and the other one only with bone, referred to as control side, were included in this evaluation. The gender distribution was 15 (65.2%) female and 8 (34.7%) male patients. The age range of the patients was 30–90 years. Altogether 171 dental implants were inserted, 90 implants in the PRP and 81 in the control group. The implant systems used were: Xive^®^ implants (Dentsply Sirona, York, USA), Straumann^®^ Standard Plus (Straumann, Basel, Swizerland), Brånemark MK III TiUnite^®^ Implants (Nobel Biocare, Kloten, Switzerland), and Osseotite^®^ implants (Biomet 3i, Munich, Germany) (Figure 3). The average time between the implant insertion and follow-up examination was 13 years. Since the clinical parameters of the seven already-lost implants could not be determined, the number of implants evaluated decreased from *n* = 171 to *n* = 164.

#### 3.1.1. Plaque index

Table 1 shows the results of the clinical examination for plaque accumulation on the implants. On average, a plaque index of 1 on the PRP and 1.2 on the control side was documented. Fisher’s exact test (*p* = 0.57) showed no statistically significant difference between the PRP and the control side.

#### 3.1.2. Probing Depth

The maximum probing depth measured on each implant was recorded (Table 2). On average, the maximum probing depths were 4.1mm (Standard Deviation (SD) 1.3) on the PRP side and 3.8 mm (SD 1.2) on the control side, with a median of 4 (min.: 2, max.: 7) on both groups. The Mann-Whitney and Wilcoxon test showed no statistically significant difference between the PRP and the control side (*p* = 0.11).

#### 3.1.3. Bleeding Index

On the PRP side, 51 implants (60%) did not bleed on probing and 35 (40%) did bleed. On the control side, bleeding occurred in 22 implants (27.8%), but 57 implants (72.2%) did not bleed. There was no difference with Fisher’s exact test (*p* = 0.14) between the PRP and control sides.

#### 3.1.4. Mobility Grade

None of the examined implants in both PRP and control sides showed any signs of mobility.

#### 3.1.5. Periotest^®^ Value

Table 3 shows the Periotest^®^ measurements. The values were summarized in three value ranges. With two implants, one on the PRP and one at the control side, the Periotest^®^ measurement was more than 10. One of the implants had a loose superstructure, the other implant had no other clinical or radiographic abnormalities other than the remarkable Periotest^®^ value. On average, a Periotest^®^ value of 1.3 was determined on the PRP and the control side. Fisher’s exact test with p = 0.69 showed no statistically significant difference between the PRP and the control side.

#### 3.1.6. Alveolar Ridge Height

**Figure 4:**
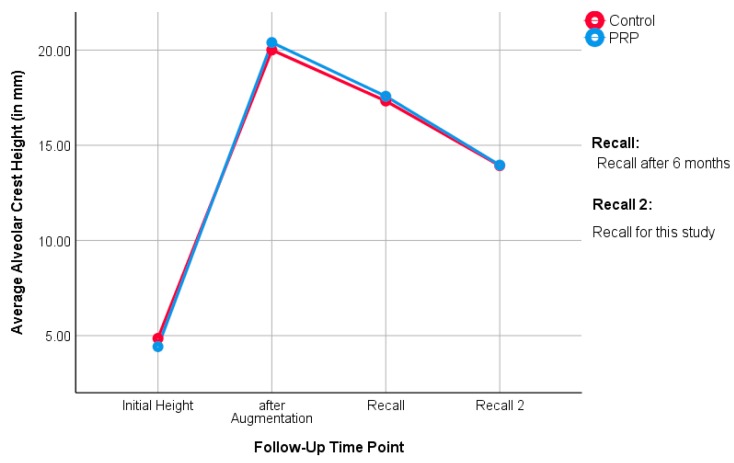
The alveolar crest height at the follow-up examination between the PRP and control side (split-mouth evaluation).

The distance from the sinus floor to the alveolar crest in the panoramic X-ray in the augmented region was measured as the height of the alveolar ridge. This ranged in the PRP side between a minimum of 9.6 mm and maximum of 21.9 mm, with a mean SD of +/- 3.4 mm and a median of 13.3 mm. In the control side, the distance ranged between minimum of 9.4 mm and maximum of 23.9 mm, with SD of 4.1 mm and median of 12.7 mm. The rank sum test according to Mann-Whitney and Wilcoxon with *p* = 0.72 indicates no difference between the PRP and the control side (Figure 4).

#### 3.1.7. Bone Loss at Augmented Areas

Absolute bone loss represents the difference in alveolar crest height after augmentation (post-operative) and measured alveolar crest height in this study (follow-up). The Mann-Whitney and Wilcoxon rank sum test showed no difference between the median bone loss values of the PRP side and the control side (*p* = 0.88). The percentage of bone loss represents the ratio of absolute bone loss to bone height after augmentation. There was no significant difference between the two sides with the rank sum test according to Mann-Whitney and Wilcoxon (*p* = 0.53) (Table 4).

### 3.2. Unilateral Evaluation

**Figure 5:**
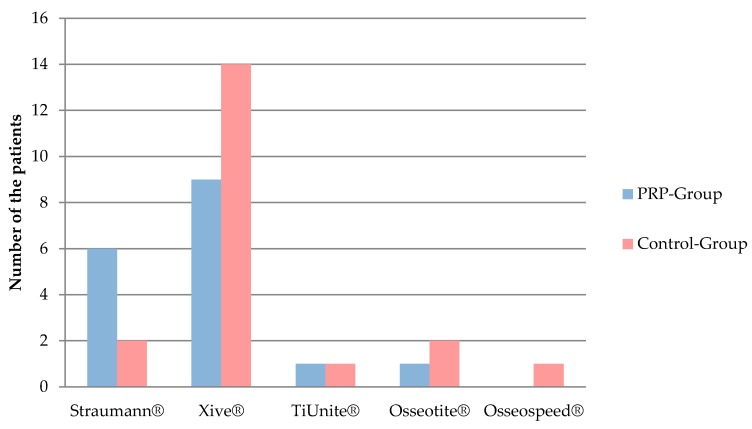
Distribution of implant systems used in PRP and control groups (unilateral evaluation).

The unilateral evaluation includes 14 unilaterally treated patients and the 23 bilaterally treated patients that were randomly included with one side of the maxilla in the evaluation, so that the participants belonged to either the PRP or the control group. In conclusion, of the 37 patients, 20 (13 female, 7 male) were in the control group and 17 patients (12 female, 5 male) belonged to the PRP group. Fischer’s exact test demonstrated the even gender distribution between the groups (*p* = 1.0). The age of the patients in the PRP group was 31–90 years with SD, median, and mean of 16.6, 81, and 60.1 years respectively. The age of the patients in the control group was 30–81 years, with SD of 15.8, median of 68, and mean of 65.9 years. The rank sum test according to Mann-Whitney and Wilcoxon with *p* = 0.26 showed that the two study groups were not different in terms of median age. Table 5 illustrates how the patients’ data on smoking behavior was distributed. Fischer’s exact test indicated *p* = 0.53 for the structural similarity of the two groups. A total of 127 implants were inserted. Of these, 58 implants (45.7%) were placed in the PRP group and 69 implants (54.3%) in the control group. The homogeneity of the two groups was proven by using Fisher’s exact test (*p* = 0.53) with respect to the number of implants per patient. Figure 5 shows the distribution of the implant systems used in the two groups. The structural similarity between the groups was proven by Fischer’s exact test (*p* = 0.37). Time between implant placement and follow-up examination in the PRP group was between 11.6 and 14.6 years, with a mean of 13.0 (SD 0.9) years and median of 13 years. In the control group, the follow-up time ranged between 11.3 and 15.1 years, with a mean of 12.9 (SD 1.1) years and median of 13 years. The rank sum test according to Mann-Whitney and Wilcoxon with *p* = 0.96 proved the structural similarity between the PRP and the control groups. Due to the four implants lost, the number of implants was reduced from *n* = 127 to *n* = 123 in the evaluation of clinical and radiological parameters.

#### 3.2.1. Plaque Index

Table 6 shows the distribution of the Mombelli plaque index in the PRP and control groups. On average, the plaque index was found to be 0.9 in the PRP group and 1.1 in the control group. Fisher’s exact test demonstrated no difference between the two groups (*p* = 0.28).

#### 3.2.2. Probing Depth

Table 7 shows the distribution of the maximum probing depths measured on the implants in the PRP and control groups. The average probing depth for the PRP group was 4.2 mm (SD 1.37), and 3.6 mm (SD 1.06) for the control group (median PRP: 4, range: 2–8; median control: 3, range: 2–7). The Mann-Whitney and Wilcoxon test of *p* = 0.005 proved a statistically significant difference between the two groups.

#### 3.2.3. Bleeding Index

Peri-implant tissue bled upon probing in 22 implants (40%) of the PRP group, 33 (60%) did not bleed. In the control group, bleeding occurred in 19 implants (27.9%), and did not occur in 49 (72.1%). Fischer’s exact test could not prove any statistically significant difference between the two groups (*p* = 0.18).

#### 3.2.4. Mobility grade

All examined implants had a degree of loosening of 0.

#### 3.2.5. Periotest^®^ value

Table 8 gives an overview of the distribution of recorded Periotest^®^ values. The values are displayed in value ranges. On average, a Periotest^®^ value of 1.3 was measured in the PRP group, 1.2 in the control group. Fischer’s exact test with *p* = 0.84 showed no statistically significant difference between the PRP and the control group.

#### 3.2.6. Alveolar Ridge Height

**Figure 6:**
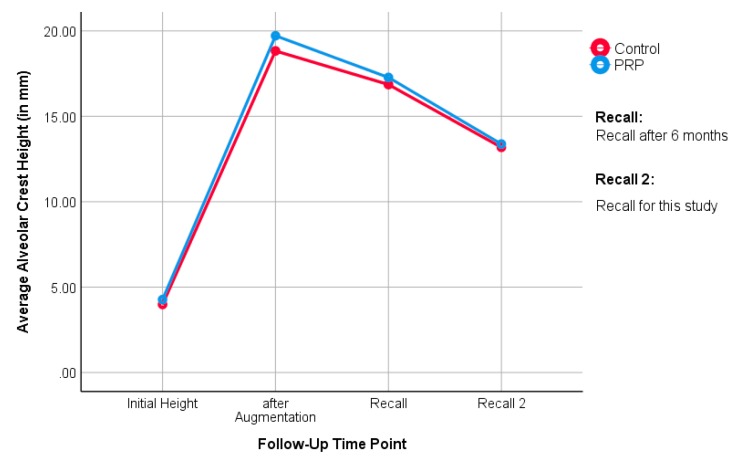
The alveolar crest height at the follow-up examination between the PRP and control sides (unilateral evaluation).

The distance from the sinus floor to the alveolar ridge was measured by a panoramic X-ray in the augmented region. The distance in the PRP group ranged between 7.1 to 21.9 mm, with SD of 4.2 and a median of 12.8. The alveolar ridge height in the control group ranged between 8 and 23.9 mm, with SD of 3.2 and median of 12.8 mm. The rank sum test according to Mann-Whitney and Wilcoxon did not prove any statistically significant difference between the PRP and the control groups (*p* = 0.96). Figure 6 shows mean alveolar crest height over time from the beginning of the previous study to the follow-up in this study.

#### 3.2.7. Bone Loss of the Augmented Region

The absolute bone loss, calculated from the difference in alveolar crest height after augmentation, and the measured alveolar crest height in the course of this study were recorded.

A comparison of the median bone loss values of the PRP and control groups with the Mann-Whitney and Wilcoxon rank sum test failed to show any difference between the two groups with *p* = 0.46.

The ratio of absolute bone loss to bone height after augmentation was calculated as percentage of bone loss. There was no difference between the PRP and the control group with the Mann-Whitney and Wilcoxon rank sum test (*p* = 0.70) (Table 9).

### 3.3. Overall Evaluation

Table 10 presents the clinical and radiological parameters (*n* = 14) of the split-mouth and the unilateral evaluation in a general overview. Overall, the values of the PRP group exceeded the control group in three parameters. The values of the control group exceeded the values of the PRP group in nine parameters. For two parameters, the values of both groups were the same. 

## 4. Discussion

This study aimed to investigate the long-term impact of PRP on the clinical and radiological treatment outcomes of dental implants. A comparison to the treatment outcome without PRP was established by the evaluation of a control group or a control side. The distribution of the patients to the study groups was randomized and the investigators were blinded (patients’ group affiliation was unknown to the investigator). This study represents a non-experimental therapeutic study with retrospective data collection and thus shows a lower level of evidence compared to other prospective studies. Nevertheless, it must be emphasized that there is currently no controlled study reporting the influence of PRP on long-term clinical and radiological outcomes, therefore, this study provides important information in this regard. The randomization and stratification of the previous trial achieved a structural equality within the individual groups. Since the present study is based on the same patient collection, structural equality, despite the drop-outs, was maintained. Additionally, this study was designed as a bicentric study, as patients from both Giessen and Erlangen were examined. Using several centers increased the external validity and improved the comparability of the results of the study [32]. One limitation of this study is the number of 37 included patients. Statistically, this represents a small population of patients. A greater quantity of patients allows for higher statistical power and can be better communicated to the general public [33]. However, as this is a long-term follow-up study in which patient population was dependent on the previous study, it is obvious that contact would not be possible for several patients.

The clinical and radiological implant parameters were hard to compare with the current literature, as there is a lack of equivalent research already published. Therefore, studies disregarding PRP were considered and contrasted with the results of the control group. The plaque index according to Mombelli et al. [30] was recorded in all investigated implants. In the PRP group, 75.3% (split-mouth evaluation) and 78.2% (unilateral evaluation) of the implants showed no or low plaque accumulation (Grade 0 or 1). In the control group, similar results were reported in 65.8% (split-mouth evaluation) and 72.1% (unilateral evaluation) of the implants. This suggests that good oral hygiene was maintained by the majority of patients in both groups. Moderate to massive plaque (Grade 2 or 3) was found in 24.7% (split-mouth evaluation) and 21.8% (unilateral evaluation) of implants in the PRP group, and 34.2% (split-mouth evaluation) and 27.9% (unilateral evaluation) in the control group. There was no significant difference between the groups. Average plaque index was 1 in the PRP group (split-mouth evaluation) and 0.9 (unilateral evaluation) and in the control group it was 1.2 (split-mouth evaluation) or 1.1 (unilateral evaluation). The implants’ plaque index in the study by Attia et al. reported Grade 0 or 1 in 75.2% of cases, while 24.8% of the implants were Grade 2 or 3 [34]. This corresponds to the results of the control group from the present study. Simonis et al. investigated 131 implants after an observation period of 10 to 16 years [35]. The average plaque index documented in this study was 1.1, which is comparable with the data of the control group from the present results.

On average, the measured pocket depth in the PRP group was 4.1 mm and 4.2 mm, and in the control group 3.8 mm and 3.6 mm in split-mouth evaluation and unilateral evaluation, respectively. Probing depths of more than 4 mm were considered pathological for implants [36]. In the present study, 67.1% (split-mouth evaluation) and 63.6% (unilateral evaluation) of the implants had probing depths of up to 4 mm in the PRP group. In the control group, a probing depth exceeding 4 mm was present in 79.8% (split-mouth evaluation) and 88.2% (unilateral evaluation) of implants. Pathological pocket depths of more than 4 mm were documented in the PRP group in 32.9% (split-mouth evaluation) and 36.4% (unilateral evaluation) of total implants, in the control group in 20.2% (split-mouth evaluation) and 11.8% (unilateral evaluation) of total implants. No statistically significant differences between the groups could be demonstrated in the split-mouth analysis with respect to the probing depths. In the unilateral evaluation, however, the PRP group had significantly higher probing depths. Here, it must be taken into consideration that the measurement of probing depths due to pseudo pockets and the design of the crown can be falsified. In case of pseudo pockets, there is no equivalent marginal bone loss despite increased probing depth. In the evaluation of crestal bone loss, no significant difference between the groups was detected in the unilateral evaluation. It can therefore be assumed that the results of the probing depths were worsened by pseudo pockets. In the study by Attia et al., 84.1% of the implants had probing depths of up to 4 mm. Pocket depths of more than 4 mm were measured on 15.7% of all implants. These values confirm the results from the control group of this study [34]. In addition, Van Velzen et al. reported in their prospective study of 177 patients probing depths of 3.7 mm with 374 implants after 10 years. This result is also comparable with the results of the control group in this study [37].

In both end points of the PRP group, 60% of implants showed no bleeding on probing. In the control group, 72% of implants did not bleed in both evaluations. No significant difference was found between the two study groups. Becker et al. examined 388 implants after an average of 14 years. They were clinically unable to detect bleeding in 79.1% of the implants. This result is similar to the values of the control group from this study [38].

No implant loosened at the time of follow-up. Thus, there is no difference between the PRP and the control group with respect to the degree of loosening. Other studies in the literature indicate the same results [34,39].

Periotest^®^ values of −8 to 0 indicate a satisfactory osseointegration of the tested implant. Values of up to 9 should be clinically re-evaluated and values of 10 and above indicate a lack of osseointegration [40]. In the present study, values ranging from -8 to 0 (68.3 to 76.5%) were observed in both groups in approximately half of the implants. Between 23.5% and 30.4% of the implants, the Periotest^®^ device delivered values of 1 to 9. Values above 10 were only determined for a maximum of 1.3% of the implants. In the two evaluations, no statistically significant difference could be detected between the PRP and the control group. Periotest^®^ measurements in this study are not very objective, however, because the supra constructions were not removed. Due to the similar study design, a comparison is merited with the results of Attia et al., who investigated implants in hypodontia patients, and they state that only one of the 148 implants tested had a Periotest^®^ value greater than 10 [34]. This corresponds to 0.7%, which is consistent with the values of the control group in the present work.

The median alveolar crest height in the PRP group was 13.3 mm (split-mouth evaluation) and 12.8 mm (unilateral evaluation), while it was 12.7 mm (split-mouth evaluation) and 12.8 mm (unilateral evaluation) in the control group. Here, it can be seen that the PRP group had slightly more bone height than the control group, until the first follow-up in the study of Schaaf et al. [19,20]. Over time, the groups ended up at equal heights. This is also noticeable when looking at the medians: in the split-mouth evaluation, the medians of the study groups are close to each other. In the unilateral evaluation, they are even identical. This suggests that the influence of PRP on the bone level of the augmented region is, if present, a short-term result. No statistical differences between the groups could be determined in the long-term follow-up.

The percent of bone loss of the augmented region relates absolute bone loss to the baseline. As a result, the percentage change is a meaningful measure for the resorption of the augmented jaw region. The median percentage of bone loss in the PRP group was 31.5% (split-mouth evaluation) and 35% (unilateral evaluation). In the control group, the values were 25.6% (split-mouth evaluation) and 27.2% (unilateral evaluation). The statistical analysis did not show any significant difference between the groups. Schmitt et al. examined 25 patients who had received augmentations in the upper jaw. After ten years, the authors found that 30.2% of augmented bone was resorbed. These findings can be regarded as equivalent to this study [41].

The majority of the clinical and radiological parameters did not show any difference between the PRP group and the control group. Thus, the hypothesis defined in this study could not be proven. Most of these results correspond to current literature and follow the findings of the previous study by Schaaf et al. [19,20]. In the same patient collection, there was no positive effect of PRP in the use of autologous bone grafts at the upper jaw in the short-term outcomes. Now, in this long-term clinical and radiological follow-up, no change could be observed, either. 

Ample evidence on the use of regenerative therapy approaches are present in the literature, most of which circumvent one or more limitation of autologous bone grafts and PRP enhancement. Nonetheless, many, if not all, alternative methods open other issues in terms of ethics, feasibility, and specialization. 

Using scaffolds in their various forms, decellularized matrix, hydrogels, or spun collagens have certain advantages in handling, and there is the possibility of loading them with mesenchymal stem cells (MSCs). Such scaffolds have shown enhanced osteogenic properties in vitro and enhanced differentiation of loaded stem cells [27]. In dental treatment, the recent discovery of periapical cyst-mesenchymal stem cells will advance this concept as a local source for stem cells [42]. To benefit from the scaffolds properties, such as porosity and low coast, osteoconductive rather than osteoinductive approaches have been addressed, especially in disturbed bone healing in cases of tumorous bone such as multiple myeloma [43], or systemically diseased bone such as in osteoporosis [44]. Different compounds could enhance osteoblasts or inhibit osteoclasts in vitro such as Donepezil [45]. The use of minerals such as calcium phosphate cements doped with active ingredients such as strontium [46] or nanoparticles loaded on scaffolds such as nanosilicates poly(glycerol sebacate) [13], showed enhanced mineralization in vivo and in vitro.

There are new upcoming development towards smart biomaterials and a faster implementation and integration of materials in patient treatment by modulating the immune response [47,48]. The main idea is to implement biomaterials to regulate inflammatory response, one of the major challenges in tissue grafts and an often neglected effect by clinicians. The future aim is to use the immune reaction to manage drug release temporarily and locally according to the need [49]. One of the methods to regulate the immune response is the regulation of oxidative stress, which is involved in the response to pathogen infection and inflammation [50,51]. However, these procedures are still far from daily clinical applications and are costly for the patient.

This paper aimed to fairly assess bone augmentation with PRP after a long-term follow-up, bone augmentation having been the state-of-the-art approach 20 years ago. Besides there being a long way until becoming a certified product, all the above-mentioned technologies are still in the early stages of development with variable unknown factors. Further long-term follow-up studies will be necessary to prove their validity.

## 5. Conclusions

Long-term clinical and radiological follow-up of dental implants inserted in autologously augmented maxillary bone was proven satisfactory, with stable results after an average of 13 years. No statistically relevant differences between the PRP and control groups regarding peri-implant parameters or remnant bone height of the alveolar ridge could be observed. Despite an expectable significant drop-out rate in this retrospective study after 13 years, the long-term results confirm the previously published RCT short-term results.

Since PRP, as an autologous adjuvant for bone regeneration, did not lead to promising results in comparison to standard surgery control groups, new approaches should be investigated. Decellularized bone, extracellular matrix, and human dental pulp stem cells as a construct for bone regeneration need to be tested in future randomized prospective clinical trials.

## Figures and Tables

**Table 1:** Distribution of the plaque index on the implants (split-mouth evaluation).

Plaque Index According to Mombelli	Platelet-rich plasma (PRP) Side		Control Side	
	Number of Implants	Percent	Number of Implants	Percent
Grade 0: No Plaque	26	30.6%	19	24%
Grade 1: Plaque visible by probing	38	44.7%	33	41.8%
Grade 2: Visible plaque accumulation	17	20%	21	26.6%
Grade 3: Massive plaque accumulation	4	4.7%	6	7.6%
Total	85	100%	79	100%

**Table 2:** Distribution of the maximum probing depth in both the PRP and control sides (split-mouth evaluation).

Maximum Probing Depth	PRP Side		Control Side	
	Number of Implants	Percent	Number of Implants	Percent
2 mm	8	9.4%	7	8.9%
3 mm	22	25.9%	29	36.7%
4 mm	27	31.8%	27	34.2%
5 mm	11	12.9%	8	10.1%
6 mm	16	18.8%	5	6.3%
7 mm	1	1.2%	3	3.8%
Total	85	100%	79	100%

**Table 3:** Distribution of Periotest^®^ value ranges (split-mouth evaluation).

Periotest^®^	PRP Side		Control Side	
	Number of Implants	Percent	Number of Implants	Percent
Values between −8 and 0: Satisfactory osseointegration	64	75.3%	54	68.3%
Values between 1 and 9: Clinical examination is necessary	20	23.5%	24	30.4%
Values ≥10: Insufficient osseointegration	1	1.2%	1	1.3%
Total	85	100%	79	100%

**Table 4:** Absolute bone loss of the augmented region/percentage of bone loss of the augmented region (split-mouth evaluation).

Patients.	PRP Side		Control Side	
Number of Patients	23		23	
Mean	6.4391 mm	29.6%	6.0783 mm	27.3%
Standard deviation	4.48135 mm	13.5%	5.49545 mm	18%
Minimum	1.30 mm	8.6%	0.40 mm	2.5%
Median	5.4000 mm	31.5%	4.6000 mm	25.6%
Maximum	19.30 mm	64.3%	21.30 mm	66.6%

**Table 5:** Distribution of smoking behavior in PRP and control groups (unilateral evaluation).

Smoking Behavior	PRP Group		Control Group	
	Number of Patients	Percent	Number of Patients	Percent
Non-smoker	12	70.6%	15	75%
Smoker and ex-smoker	5	29.4%	5	25.0%
Total	17	100%	20	100%

**Table 6:** Distribution of the plaque index on the implants (unilateral evaluation).

Plaque Index According to Mombelli	PRP Side		Control Side	
	Number of Implants	Percent	Number of Implants	Percent
Grade 0: No Plaque	21	38.2%	15	22.1%
Grade 1: Plaque visible by probing	22	40%	34	50%
Grade 2: Visible plaque accumulation	10	18.2%	16	23.5%
Grade 3: massive plaque accumulation	2	3.6%	3	4.4%
Total	55	100%	68	100%

**Table 7:** Distribution of the maximum probing depth in both PRP and control groups (unilateral evaluation).

Maximum Probing Depth	PRP Side		Control Side	
	Number of Implants	Percent	Number of Implants	Percent
2 mm	5	9.1%	8	11.7%
3 mm	13	23.6%	28	41.2%
4 mm	17	30.9%	24	35.3%
5 mm	9	16.4%	3	4.4%
6 mm	9	16.4%	4	5.9%
7 mm	1	1.8%	1	1.5%
Total	1	1.8%	0	0%

**Table 8:** Distribution of Periotest^®^ value ranges (unilateral evaluation).

Periotest^®^	PRP Side		Control Side	
	Number of Implant	Percent	Number of Implant	Percent
Values between -8–0: Satisfactory osseointegration	41	74.5%	52	76.5%
Values between 1–9: Clinical examination is necessary	14	25.5%	16	23.5%
Total	55	100%	68	100%

**Table 9:** Absolute bone loss of the augmented region/percentage of bone loss of the augmented region (unilateral evaluation).

Patients	PRP Side		Control Side	
Number of patients	17		20	
Mean	6.3412 mm	31.3%	5.6450 mm	28.2%
Standard deviation	3.86831 mm	15%	4.57032 mm	16.3%
Minimum	1.30 mm	8.6%	0.40mm	2.5%
Median	5.4000 mm	35%	4.9000 mm	27.2%
Maximum	16.70 mm	60.6%	21.30 mm	66.6%

**Table 10:** Summarizing the result of the tested parameters between the PRP and control groups in both evaluations.

Parameter		PRP-Group		Control-Group	
		Value	Rating	Value	Rating
Split-Mouth Evaluation	Plaque index (mean)	1.0	+	1.2	-
	Probing depth (mean)	4.1 mm	-	3.8 mm	+
	Bleeding index	40%	-	27.8%	+
	Periotest^®^ (mean)	1.3	=	1.3	=
	Alveolar ridge height (median)	13.3 mm	+	12.7 mm	-
	Absolute bone loss (median)	5.4 mm	-	4.6 mm	+
	Percentage bone loss (median)	31.5%	-	25.6%	+
Unilateral Evaluation	Plaque index (mean)	0.9	+	1.1	-
	Probing depth (mean)	4.2 mm	-	3.6 mm	+
	Bleeding index	40%	-	27.9%	+
	Periotest^®^ (mean)	1.3	-	1.2	+
	Alveolar ridge height (median)	12.8 mm	=	12.8 mm	=
	Absolute bone loss (median)	5.4 mm	-	4.9 mm	+
	Percentage bone loss (median)	35%	-	27.2%	+

+: value is better than in the other group, -: value is worse than in the other group, =: value is similar to the other group.
